# Letter to the editor for the article "Auto-injector needle length may be inadequate to deliver epinephrine intramuscularly in women with confirmed food allergy"

**DOI:** 10.1186/1710-1492-10-54

**Published:** 2014-11-05

**Authors:** T Ted Song

**Affiliations:** Division of Allergy and Infectious Diseases, University of Washington, School of Medicine, Seattle, WA USA

**Keywords:** Anaphylaxis, Epinephrine, Auto-injector, Needle length

## Abstract

Letter to the Editor for "Auto-injector needle length may be inadequate to deliver epinephrine intramuscularly in women with confirmed food allergy" by Tsai et al. There are limitations of this study note mentioning such as method of compression, role of propulsion, defining those patients who are at risk of prophylaxis and future studies.

## To the Editor

I read with interest the article "Auto-injector needle length may be inadequate to deliver epinephrine intramuscularly in women with confirmed food allergy" by Tsai et al. [1]. The authors report findings from a study assessing adequacy of epinephrine needle length by measuring muscle depth at the recommended site of injection for adult males and females. There are some limitations in the study worth noting.

Tsai et al. noted that this is the first study to exam needle length delivery in patients who are "at risk for anaphylaxis". This is in alignment with the European Academy of Allergy, Asthma and Clinical Immunology (EAACI) public declaration on food allergy and anaphylaxis in that they recommend that all patients at risk for anaphylaxis should carry epinephrine auto-injector [2]. EAACI recommend that epinephrine auto-injectors be available in public places since there are patients who may have first episode of anaphylaxis and do not have epinephrine auto-injectors available to them. However, do patients at risk for anaphylaxis have different weight, height or BMI, body mass index, profile than those without risk for anaphylaxis? I am not aware of any current published literature that answers this question. Until that has been studied, physicians have to recommend epinephrine auto-injectors to the general population with varying BMI’s and not to those only at risk of anaphylaxis.

I applaud Tsai et al. for considering compression in their study, which is one of the factors that play a role in ensuring delivery of epinephrine to the intramuscular space. In their study, one physician applied an ultrasound probe with maximum pressure as defined as "pressure mimicking that required for proper auto-injection". The mean change in skin-to-muscle depth (STMD) with pressure was 11% for all subjects and 16% for women. In a pilot study that we published in 2005, there was 19% reduction in STMD in males and 25% in females [3]. In our study, we placed an 8 lb weight block on top of the EpiPen® auto-injector trainer to simulate the compression in a patient who was laying lateral recumbent. I agree with Tsai et al. that the surface area of the auto-injector is different from the ultrasound transducer. Yet, I believe that the smaller surface area of EpiPen® compared to ultrasound transducer tip has a significant role in human studies. The small surface area can increase compression of the subcutaneous tissue to deliver epinephrine beyond the length of the needle-length. The difference in STMD with pressure in the two studies is most likely due to surface area of EpiPen® (Song et al.) versus ultrasound transducer (Tsai et al.). The other factors that are not addressed in this study that effect the delivery of epinephrine into the intramuscular space are spring tensile strength, force of injection, and bore diameter.

Another factor that is crucial to proper delivery of epinephrine is the propulsion of the auto-injector. Almost all the epinephrine auto-injectors are spring loaded. In other words, there is a spring that is tightly coiled and has propulsion force. We published an in vivo study in pig thighs which shows that the STMD was 2.78 ± 0.59 cm (range, 2–4 cm) with p = <0.0001 (one *sample t-test*) [4]. The STMD delivery of epinephrine was 94.4% beyond the length of the auto-injector needle. The authors believe that compression, or hand swing, and propulsion by the spring loaded auto-injector, have a role in delivery of epinephrine beyond the needle length. In the United Kingdom, there is an epinephrine auto-injector, Emerade®, which has a needle length of 2.5 cm. Tsai et al. believe that at this needle length, 96% of their female patients would receive epinephrine intramuscularly. I agree that the needle length of 2.5 cm would be helpful in many female patients with high BMI but it may be too long for some males with normal to overweight BMI. Further in vivo studies considering compression of tissue and propulsion, dispersion of epinephrine beyond needle tip, and the ideal needle length would be helpful.

In addition, future studies in human models, stratifying patients based on BMI would be helpful. I look forward to a table with gender, BMI and STMD so that we can recommend a proper needle length for our patients who are at risk of anaphylaxis.

## Authors’ response

Harold Kim, Sten Dreborg, Laura Kim and Gina Tsai

The authors’ reply: Song raises a number of important questions regarding some potential limitations of our study "Auto-injector needle length may be inadequate to deliver epinephrine intramuscularly in women with confirmed food allergy". (1) We will address each issue.

The first concern is whether patients at risk for anaphylaxis have different weight, height or BMI then patients who are not at risk for anaphylaxis. As far as we are aware, Song’s comments are correct in that there is no data comparing these variables in adults with and without food allergy. In our study, we felt that it seemed most logical to study the population at risk of anaphylaxis. The mean BMI for all subjects in our study was 25 kg/m2. (1) Data from a general census in the same municipality where the study was completed showed that 53.9% of adults were either overweight or obese in 2011-2012. Unfortunately, the mean BMI was not reported in the census.(2) So we are uncertain of the differences of the BMI of our subjects with food allergy and the general population. As well, the range of BMI in our study was quite wide at 18 to 50 kg/m2 and although the BMI correlated with STMD it was not a perfect predictor of adequate STMD for the epinephrine auto-injectors. We concluded in our paper that patients at risk of anaphylaxis and especially women should have an ultrasound of the mid-anterior thigh completed to determine if the STMD is adequate for an epinephrine auto-injector.

In our study, we attempted to simulate the STMD distance when an epinephrine auto-injector is delivered by performing our ultrasound measurements with "maximal" pressure in the mid anterolateral thigh. Certainly, this technique is not perfect. Optimally, an ultrasound probe with the exact surface area of each epinephrine auto-injector pushed down at the exact pressure used to trigger the auto-injector should be used to measure the STMD. But as noted in our paper, even if we applied the exact pressure that is required to trigger the auto-injectors, it will not be an indicator of the exact pressure used when the auto-injector is used in real life. So we used "maximum" pressure at the appropriate site for epinephrine injection. We felt that with this technique we would be estimating the STMD that would be attained with the auto-injectors in the "best case scenario" for adults. Song references his paper showing a 19% reduction in STMD in males and a 25% reduction in females using an 8 pound weight for compression. (3) These measurements were completed on one male and one female only. We do not believe that this is the best approach to estimate compression in all subjects. In fact, in our paper the percent change in STMD with and without "maximal" pressure or compression ranged from 0 to 45% with a mean of 11%. Again, we would recommend performing ultrasound measurements in all subjects at risk of anaphylaxis with and without pressure. As well, future studies are required to assess tissue compression with more accurate models simulating epinephrine auto-injector use. A third concern raised by Song is that the STMD may underestimate the true delivery depth of the epinephrine. He points out that the epinephrine would be delivered deeper than the tip of the needle because the auto-injectors are spring-loaded leading to a propulsion force and deeper delivery of the medication. In his study, using pig thighs, it was shown that the delivery of epinephrine was 94.4% deeper than the needle tip. (4) This data is interesting and may be true for the injection within one tissue compartment. But a study presented this year at the European allergy meeting showed that the epinephrine delivery would stay in the subcutaneous space if the needle tip ended in the subcutaneous space. More specifically, the needle tip would have to penetrate the muscle fascia to deliver the epinephrine intramuscularly. (5) Therefore, we stand by our suggestion that we require longer needle lengths for some subjects where the STMD is greater than the needle lengths of the currently available auto-injectors. As well, Song raised a concern that a 2.5 cm needle length may be too long for some male subjects. We agree that this is a may be a possibility in very few subjects with thin thighs. Epinephrine auto-injectors with the appropriate needle length that require a lower pressure for injection may be required in these subjects.

In summary, we agree that future research correlating variables such as gender, BMI and STMD would be helpful in subjects at risk of anaphylaxis. But we believe that a "personalized" medicine approach with the use of ultrasound and then recommending the auto-injectors with the appropriate needle length and dose of epinephrine is the best clinical approach. Finally, patients at risk of anaphylaxis should have two auto-injectors available and they should seek medical attention immediately if a auto-injector(s) is required.

## Abbreviations

BMI: Body mass index
STMD: Skin-to-muscle depth.
